# Dr. Krishna Manohar Soman Rema: A Legacy of Innovation, Education, and Compassion in Pediatric Cardiac Surgery

**DOI:** 10.7759/cureus.78426

**Published:** 2025-02-03

**Authors:** Sowmya Ramanan, Krishnan Ganapathy Subramaniam, Baiju Sasi Dharan, Valikapathalil Mathew Kurian, Vishal V Bhende

**Affiliations:** 1 Pediatric Cardiac Surgery, Sree Chitra Tirunal Institute for Medical Sciences and Technology (SCTIMST), Thiruvananthapuram, IND; 2 Pediatric Cardiac Surgery, Sri Padmavati Pediatric Heart Centre, Sri Venkateswara Institute of Medical Sciences (SVIMS) Campus, Tirupati, IND; 3 Cardiothoracic and Vascular Surgery, Institute of Cardiovascular Diseases (ICVD) Madras Medical Mission, Chennai, IND; 4 Pediatric Cardiac Surgery, Bhanubhai and Madhuben Patel Cardiac Centre, Shree Krishna Hospital, Bhaikaka University, Karamsad, IND

**Keywords:** affordable healthcare, biography, cardiothoracic surgery, historical vignette, krishna manohar sr, sri sathya sai sanjeevani hospital

## Abstract

Dr. Krishna Manohar Soman Rema (SR) (1956-2022) was a pioneering congenital cardiac surgeon and an inspiring educator who dedicated his life to advancing pediatric cardiac care and mentoring the next generation of surgeons. Trained in cardiac surgery at the Sree Chitra Tirunal Institute for Medical Sciences and Technology in Kerala, he served as faculty there for 20 years, contributing significantly to the development of the Chitra Heart Valve. His career also included tenures at the Madras Medical Mission, Frontier Lifeline Hospital, and the Sri Sathya Sai Hospital in Whitefield, Bengaluru. Dr. Krishna Manohar’s most enduring contribution was his role in establishing cardiac surgical services at the Sri Sathya Sai Sanjeevani International Centre for Child Heart Care and Research in Palwal, Haryana, part of a network of hospitals offering free congenital heart surgery to underprivileged children. Under his leadership, these centers performed over 22,000 open-heart and interventional procedures over a decade, achieving excellent outcomes. An exceptional mentor, Dr. Krishna Manohar introduced a transformative *ABCD* framework for cardiac surgery, emphasizing adaptability, befriending collaboration, compassion, and discipline. He employed innovative teaching methods, such as the *Handicraft Your Own Heart* technique, to simplify complex concepts for trainees. Recognized for his dedication, he received numerous accolades, including the Lifetime Achievement Award from the Pediatric Cardiac Society of India in 2023. Dr. Krishna Manohar’s legacy lives on through his students and the institutions he helped build, inspiring future generations to continue his mission of providing high-quality, accessible care to children with congenital heart disease (CHD).

## Introduction and background

This review highlights the significant contributions of Dr. Krishna Manohar Soman Rema (SR) (1956-2022), an Indian cardiothoracic surgeon, in improving access to healthcare for children with congenital heart disease (CHD) in India. It also underscores his legacy as a passionate educator, which continues after his death by donating his remains to the Department of Anatomy at Government Medical College, Thiruvananthapuram [1,2].

## Review

Early life and education

**Figure 1:**
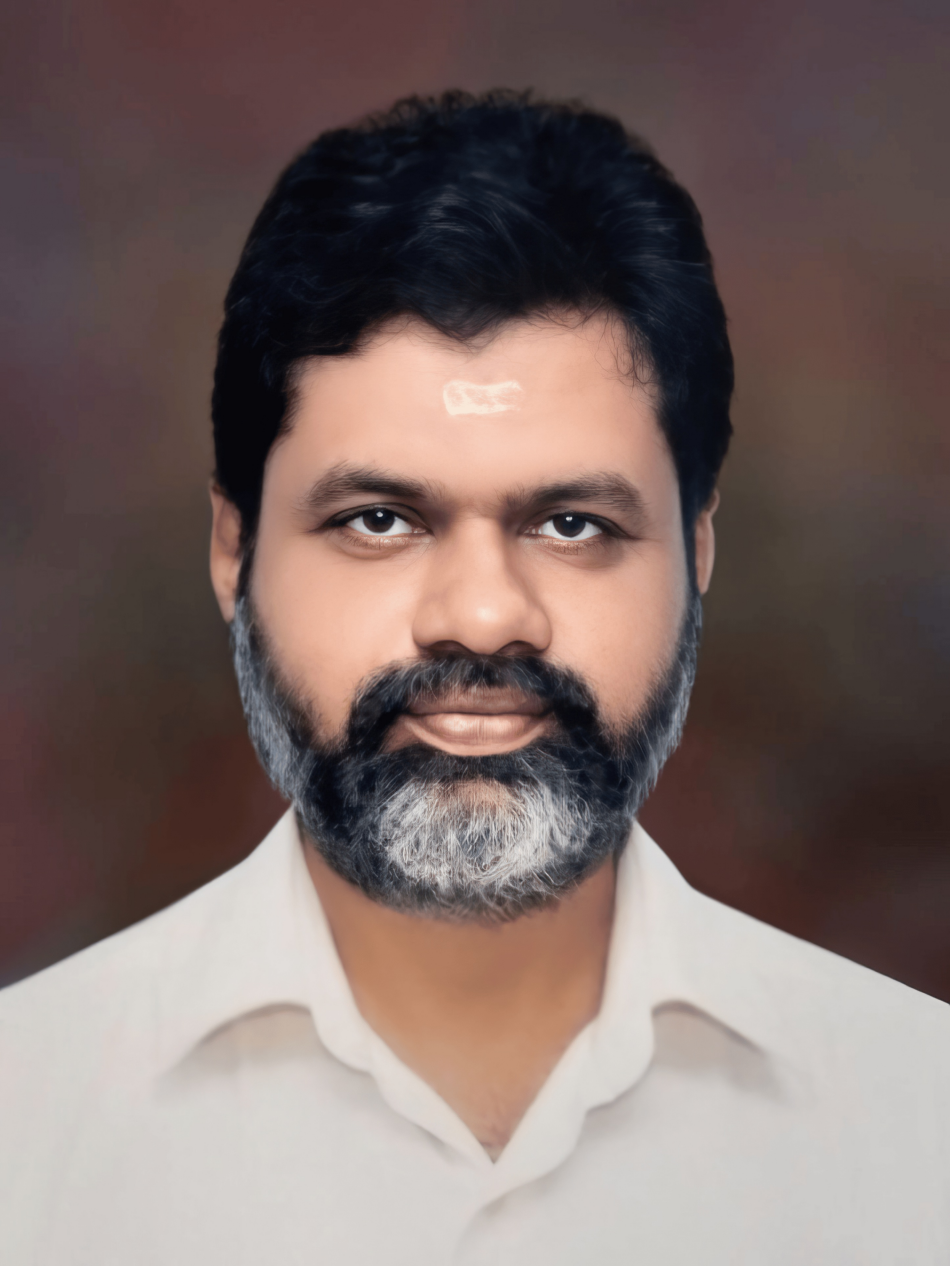
Dr. Krishna Manohar Soman Rema (1956-2022): a life devoted to congenital cardiac surgery. Image credit: The photo was taken from the personal collection of Dr. Krishna Manohar with the permission of the family.

Dr. Krishna Manohar SR was born on May 24, 1956, in Anchal, a village in Kerala, India (Figure 1). After completing his early education in Kollam, Kerala, he joined Government Medical College, Thiruvananthapuram, in 1979 and later trained in general surgery at Government Medical College, Kozhikode, in 1985. His early exposure to medicine came through assisting his traditional Ayurvedic physician grandfather, which sparked his lifelong interest in healing. He married Dr. Sheila Balakrishnan, an accomplished obstetrician and gynecologist. Their son, Kiran, is an engineer and educator [1,2].

Academic pursuits and career as a congenital cardiac surgeon

Dr. Krishna Manohar’s interest in cardiac surgery began when serving as a house officer at the Sree Chitra Tirunal Institute for Medical Sciences and Technology (SCTIMST), where he worked under the mentorship of Professor M.S. Valiathan. He completed his cardiac surgery residency in 1988 and earned an MCh in cardiothoracic and vascular surgery. Known for his academic excellence, he supported his peers with their academic and personal challenges. He was an integral member of the research team developing the Chitra Heart Valve [1,2].

In 1989, Dr. Krishna Manohar joined SCTIMST as a faculty and retired in 2009 as a professor specializing in pediatric and congenital heart surgery. Notably, he was the first assistant in the historic implantation of the Chitra Heart Valve in a human patient in December 1990 [3]. During his tenure, he frequently visited the Madras Medical Mission (MMM) to observe congenital cardiac surgeries led by Dr. K.M. Cherian, further honing his expertise. After leaving SCTIMST in 2009, he joined MMM and later Frontier Lifeline Hospital, working as a senior consultant in pediatric cardiac surgery.

In 2014, following the advice of his mentor, Dr. M.S. Valiathan, Dr. Krishna Manohar joined the Sri Sathya Sai Sanjeevani Centre for Child Heart Care in Naya Raipur (now Atal Nagar, Chhattisgarh). There, he helped establish a specialty center dedicated to providing free treatment to children with CHD [3].

Books and publications

Throughout his career, Dr. Krishna Manohar authored approximately 245 papers published in Indian and internationally indexed journals. In 2016, he began documenting the history of congenital cardiac surgery in India. This effort involved extensive travel, and interviews with pioneers in the field, and culminated in a historical article, “Pioneers of Congenital Heart Surgery in India: Historical Perspective,” published in the *Indian Journal of Thoracic and Cardiovascular Surgery* on March 7, 2020 [4].

Teacher, mentor, and humanitarian

**Figure 2:**
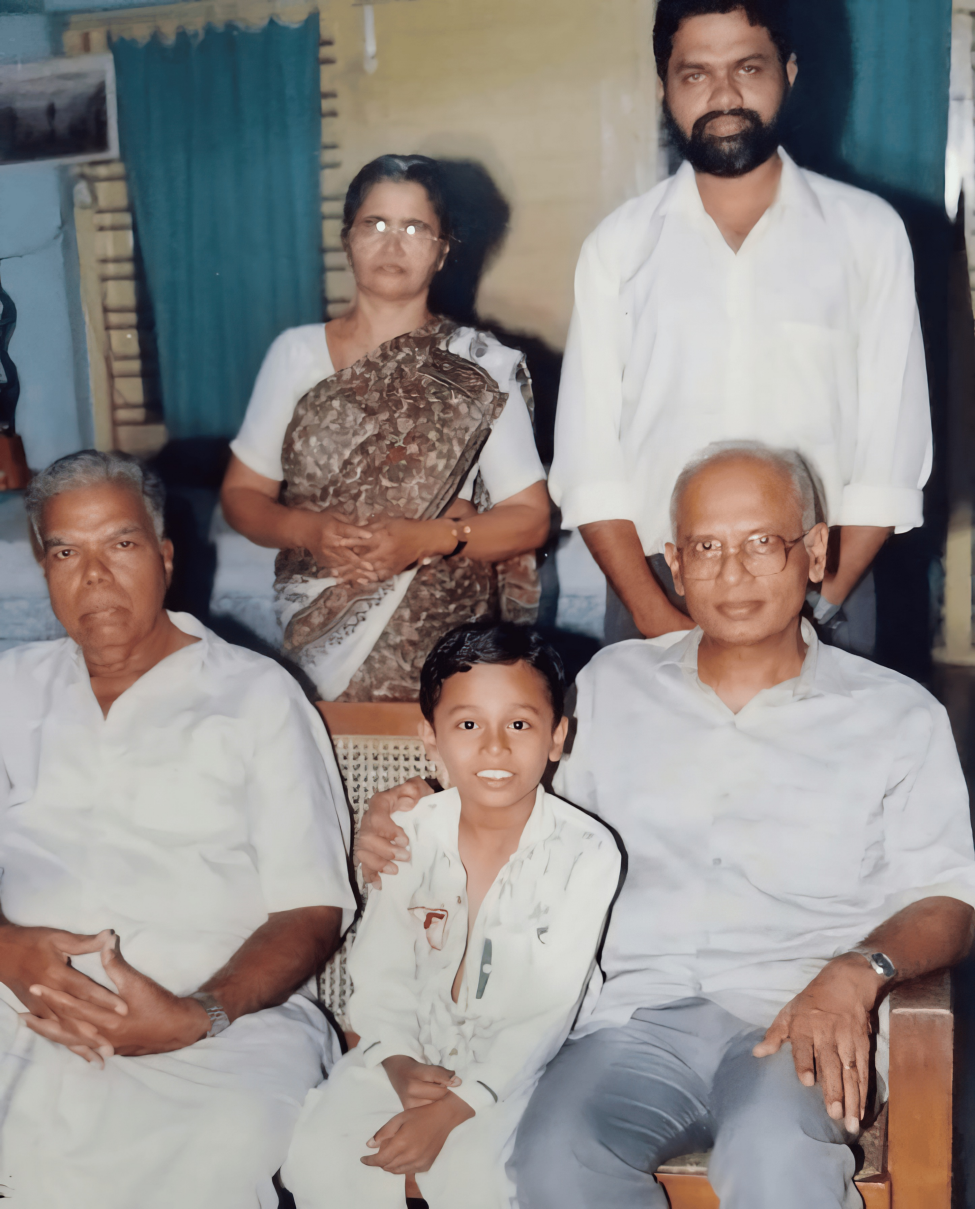
Dr. Krishna Manohar with family and his mentor (seated from left to right): A.N. Soman (father), Kiran Manohar (son), Prof. M.S. Valiathan (mentor); (standing from left to right) Rema Soman (mother) and Dr. Krishna Manohar SR in 1994. Image credit: The photo was taken from the personal collection of Dr. Krishna Manohar with the permission of the family.

Dr. Krishna Manohar exemplified the ethos of *Matha Pitha Guru Daivam* (mother, father, teacher, God) in his life and practice. He honored his teachers annually with gifts and traditional Onakkodi during Onam celebrations and personally visited them on their birthdays. Notably, he shared a birthday with his mentor, Dr. Valiathan, on May 24 (Figure 2) [1-3].

A role model for his students, he inspired many to pursue careers in pediatric cardiac surgery. His students now lead units in various institutions across India. Beyond his academic contributions, Dr. Krishna Manohar supported his students through personal challenges and fostered an environment of respect and collaboration. Patients, nurses, doctors, and staff universally admired him for his surgical expertise, dedication to patient care, humility, and willingness to help others [4].

His compassion extended beyond the operating room. The first recipient of the Chitra Heart Valve, Mr. K.D. Muralidharan, remained a close friend, with annual follow-up visits evolving into reunions celebrating the surgical milestone. Dr. Krishna Manohar also supported his community, gifting land and constructing a building for his childhood library when it faced eviction. The library was named in honor of his father as the A.N. Soman Master Public Library [4].

Free surgeries for children with CHD

Dr. Krishna Manohar’s ambition was to provide free treatment for underprivileged children in India with CHD. His work at the Sathya Sai Sanjeevani Hospital in Raipur provided the ideal environment to achieve this goal. He later moved to Palwal, Haryana, and worked at Sathya Sai Sanjeevani Hospital, Palwal, from 2018 until his death. Under his leadership, the team at Palwal performed over 4,000 surgeries on children with CHD [5].

**Figure 3:**
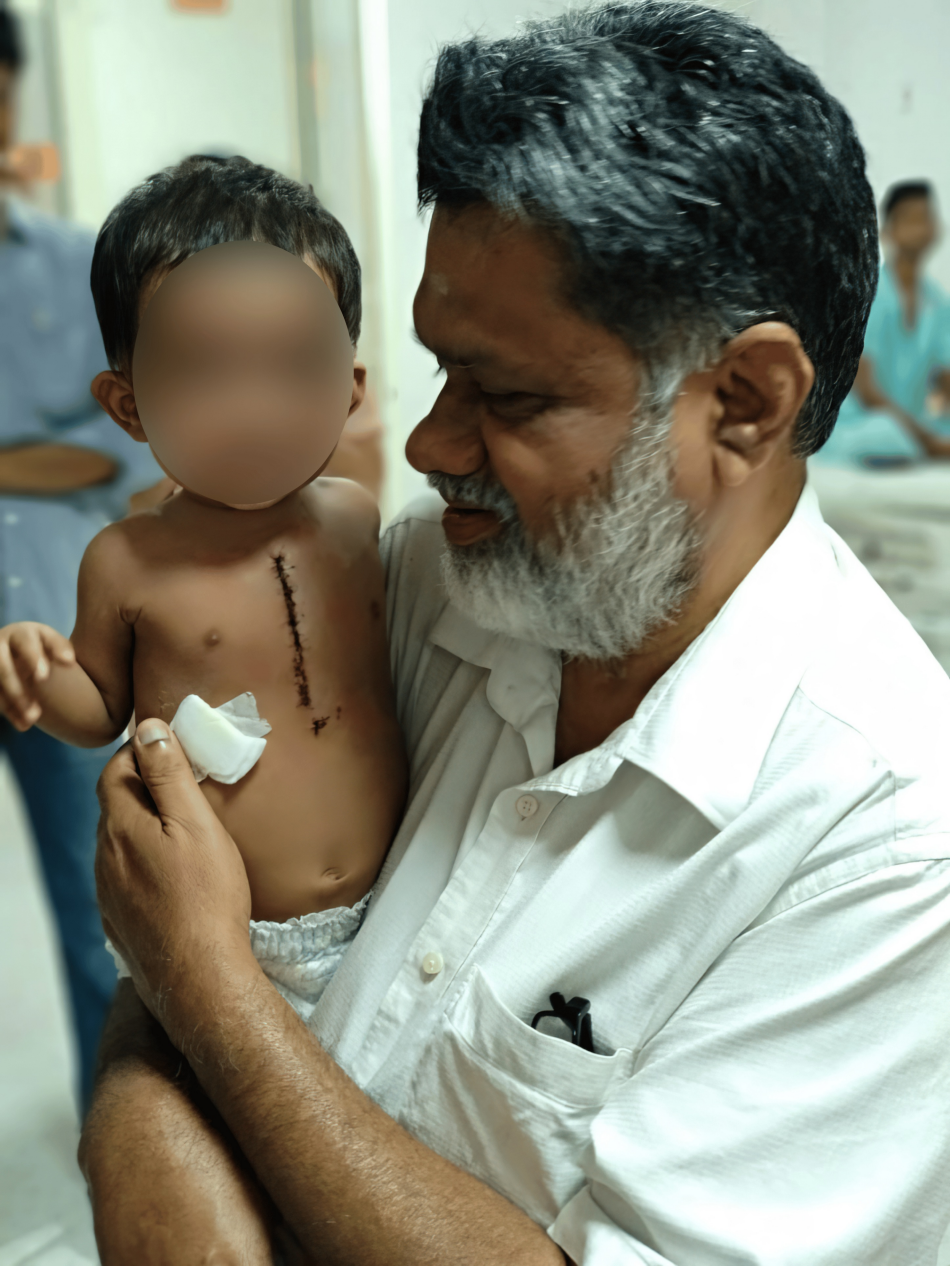
Dr. Krishna Manohar Soman Rema with one of his operated patients. Image credit: The photo was taken from the personal collection of Dr. Krishna Manohar with the permission of the family.

In 2022, Dr. Krishna Manohar’s team inaugurated services at a newly established medical college in Muddenahalli, Karnataka, performing 23 cases that year. His team also expanded their work internationally, offering services at Sri Sathya Sai Sanjeevani Children’s Hospital in Fiji and Malaysia in 2022. Over a decade ending in November 2022, the three Sathya Sai Sanjeevani hospitals collectively performed more than 22,000 open-heart and interventional procedures, primarily for complex heart anomalies, with excellent outcomes (Figure 3) [5].

Passionate teacher and pioneer in cardiothoracic surgery

Dr. Krishna Manohar was dedicated to making congenital cardiac surgery simple, safe, and reproducible. As a mentor, he inspired confidence in his trainees, including those who struggled with self-doubt and instilled a strong sense of responsibility in them. He introduced a revised *ABCD* framework of cardiac surgery to guide his students. According to this approach, surgeons should (A) adapt to and appreciate all available help; (B) befriend their colleagues, including assistants, scrub nurses, cardiologists, and management; and (C) show care and compassion to team members and patient’s families. Finally, he emphasized the importance of (D) developing a sense of duty and discipline to contribute to personal growth and the growth of others [1-4].

This philosophy enabled Dr. Krishna Manohar to build dedicated teams of cardiologists, anesthetists, and surgeons who worked together to establish free pediatric cardiac surgery centers in India and internationally under the Sri Sathya Sai Health and Education Trust, founded in May 1970. Dr. Krishna Manohar also developed and popularized numerous innovative surgical techniques, which he enthusiastically taught to young surgeons (Table 1) [6,7].

**Table 1:** Surgical innovations and techniques developed by Dr. Krishna Manohar.

No.	Surgical technique
1	Use of venous line extensions to facilitate venous cannula changes without halting cardiopulmonary bypass
2	Innominate vein cannulation for superior cavopulmonary connection, total anomalous pulmonary venous connection, sinus venosus atrial septal defect repair, atrial switch procedure, and other surgeries [6]
3	Warden procedure for repairing sinus venosus atrial septal defects
4	Iliac artery and vein cannulation for peripheral access in pediatric patients [7]
5	Septal superior approach for total anomalous pulmonary venous connection repair, creating wide anastomoses between the common chamber and left atrium
6	Single-stage ventricular septal defect and coarctation repair using thoracotomy and sternotomy
7	Repair of circumflex aorta via a median sternotomy approach

Dr. Krishna Manohar also simplified the teaching of complex topics, such as cardiac development and embryology, through innovative methods. His *Handicraft Your Own Heart* technique demonstrated cardiac anatomy using hand models, leaving a lasting impression on trainees [8]. His passion for history inspired his annual lectures on cardiac surgery’s pioneers and embryology at the MMM, which became highly anticipated events for students and trainees [9].

Awards and honors

In 2022, Government Medical College, Thiruvananthapuram, awarded Dr. Krishna Manohar a Distinguished Alumni Award during its 70th anniversary celebrations in recognition of his exceptional service to humanity and the medical profession. In 2023, the Pediatric Cardiac Society of India posthumously honored him with a Lifetime Achievement Award and named an award in his memory for the best surgical paper presentation [1,2].

## Conclusions

Dr. Krishna Manohar’s mentor and colleague, Dr. Valiathan, said that the spread of cardiac hospitals for children is “an idea whose time has come.” According to Dr. Valiathan, "This expansion continues and 'seems unstoppable' as the awareness grows that children with congenital heart anomalies can achieve good health and normal lifespans through surgery. Krishna Manohar pioneered this restorative mission with professional expertise, masterly skill, and an embodiment of goodness.” Dr. Krishna Manohar often said, “For a doctor, the patient is God. Caring for patients is the best form of worship.” His legacy exemplifies this philosophy through his exceptional contributions to CHD treatment, mentorship, and service to humanity.
